# Investigating information seeking in ravens (*Corvus corax*)

**DOI:** 10.1007/s10071-020-01372-5

**Published:** 2020-03-21

**Authors:** Megan L. Lambert, Mathias Osvath

**Affiliations:** 1 Department of Philosophy and Cognitive Science, Lund University, Lund, Sweden; 2 Comparative Cognition Unit, Messerli Research Institute, University of Veterinary Medicine Vienna, Medical University of Vienna, University of Vienna, Vienna, Austria

**Keywords:** Information seeking, Apes, Corvid cognition, Uncertainty, Metacognition

## Abstract

Measuring the responses of non-human animals to situations of uncertainty is thought to shed light on an animal’s metacognitive processes; namely, whether they monitor their own knowledge states. For example, when presented with a foraging task, great apes and macaques selectively seek information about the location of a food item when they have not seen where it was hidden, compared to when they have. We presented this same information seeking task to ravens, in which a food item was hidden in one of three containers, and subjects could either watch where the food was hidden, infer its location through visual or auditory clues, or were given no information. We found that unlike several ape species and macaques, but similar to capuchin monkeys, the ravens looked inside at least one tube on every trial, but typically only once, inside the baited tube, when they had either witnessed it being baited or could visually infer the reward’s location. In contrast, subjects looked more often within trials in which they had not witnessed the baiting or were provided with auditory cues about the reward’s location. Several potential explanations for these ceiling levels of looking are discussed, including how it may relate to the uncertainty faced by ravens when retrieving food caches.

**Electronic supplementary material:**

The online version of this article (10.1007/s10071-020-01372-5) contains supplementary material, which is available to authorized users.

## Introduction

Humans and other animals are routinely faced with situations of uncertainty in which limited information is provided, for example, about the location of food, the identity of an approaching animal or the suitability of a tool for solving a particular problem (Griffin 2003). Responding flexibly to these situations can therefore be an adaptive means of foraging, avoiding danger or solving problems more efficiently. Such responses might entail changing behaviour, opting for a more certain alternative, or seeking additional information that can facilitate a solution. These behaviours are thought to shed light on an animal’s metacognitive processes, and more specifically whether they can monitor and respond to their own uncertainty as humans do. For example, when provided with limited information about the location of a toy, 20-month old infants respond to their uncertainty by seeking additional information; namely, enlisting an adult to help them locate the toy (Goupil et al. 2016). This suggests that uncertainty monitoring develops early in humans. Studies of some non-human species show similar responses; however, whether these tasks show true metacognition in the sense that memories, for example, are represented as memories, or whether they indicate some other form of mechanism is an ongoing debate (Carruthers 2008; Hampton 2009; Jozefowiez et al. 2009; Kornell 2014; Roberts et al. 2012; Smith et al. 2012).

Thus far, several different paradigms have been used to measure aspects of metacognition in non-human animals. Often, subjects are trained to respond to a particular type of stimulus based on a rule (e.g., selecting the stimulus with the greatest number of dots, or that matches a sample they have just viewed). The trials then vary in difficulty and subjects can respond flexibly based on their degree of uncertainty. Such responses can include opting out of the trial (e.g., Hampton 2001), betting retrospectively on the certainty of their choice (e.g., Kornell et al. 2007), or exhibiting spontaneous post-trial confidence judgements through their likelihood of anticipating a reward (go-when-you-know; Beran et al. 2015). Subjects that exhibit these responses selectively on more difficult or ambiguous trials are thought to be responding based on some assessment of their own uncertainty. Alternatively, it has been proposed that subjects may have instead learned to associate particular configurations of stimuli with different responses (reviewed in Smith et al. 2012).

A second task, first introduced by Call and Carpenter (2001), addresses this by presenting subjects with a simpler foraging problem, in which they can choose to seek additional information to solve the problem. In the task’s most basic form, subjects can search for a food item hidden in one of several containers, either after witnessing where it was placed, or not. Subjects attempting to correct their lack of knowledge about the food’s location are expected to look into the containers significantly more often on trials when they had not witnessed the baiting. Indeed, both the apes (chimpanzees and orangutans) and children tested in Call and Carpenter’s original study exhibited this pattern. Further studies replicated these results with chimpanzees and orangutans and extended them to gorillas and bonobos (Call 2010; Marsh and MacDonald 2012). Similar responses have been reported for rhesus and lion-tailed macaques, which reach immediately for the correct tube after they have watched it being baited and look into the tubes significantly more when they have not (Hampton et al. 2004; Marsh 2014).

Several alternative hypotheses have since been proposed to explain differences in looking between seen and unseen trials, however (reviewed in Call 2012, see also Basile et al. 2015). Among others, it has been suggested that increased searches on unseen (also referred to as ‘hidden’) trials, where subjects did not witness the baiting, reflect a generalized search strategy such that they will search for food if none is visible, or reach for food when its location is known (Carruthers 2008; Perner 2012). For example, on hidden trials consisting of a single opaque container and two empty, transparent containers, lion-tailed macaques and capuchins continue to look into the containers despite being able to logically infer the reward’s location (Marsh 2014; Marsh et al. 2015; Vining and Marsh 2015). In contrast, apes respond flexibly, looking less on trials in which they can infer the reward’s location through auditory cues (i.e., the experimenter shakes the containers, such that the food can be heard inside the baited container) or by exclusion, and more when the reward is of higher value or the delay between baiting and selecting is greater (Call 2010; Call and Carpenter 2001; Marsh and MacDonald 2012). Additionally, some subjects continue to look in the baited containers on a small number of ‘seen’ trials, in which they have witnessed the baiting (ibid*.*). For chimpanzees and orangutans, selective information seeking also extends beyond an immediate food retrieval context, as shown by recent studies in which subjects selectively sought information about the functional properties of tools (Bohn et al. 2017; Mulcahy 2016).

A second possibility is that subjects may generally look inside the containers not because they are seeking information, but because the sight of the reward is intrinsically hedonic (Call 2012). This explanation would predict that subjects look inside the tubes on every trial, including those in which they are certain of the reward’s location. On the contrary, most primate species tested tend to look significantly less on ‘seen’ trials and/or ‘hidden’ trials in which they receive auditory or inferential–but not visual–clues about the reward’s location (Call 2010; Call and Carpenter 2001; Hampton et al. 2004; Marsh and MacDonald 2012).

Though a few additional species have been studied in uncertainty monitoring and metamemory tasks [e.g., rats, pigeons, dolphins: (Foote and Crystal 2007; Inman and Shettleworth 1999; Smith et al. 1995)], thus far, little research on information seeking exists for non-primate species. Two studies with dogs found that they did not seek information about the location of food when they had not seen where it was placed (Bräuer et al. 2004), but rather sought information from human informants (McMahon et al. 2010). A more recent study found that dogs sought information about a hidden food reward or toy more often when they had not seen which of two barriers it was placed behind and when the food was higher-value, though their search behaviour did not change with longer delays (Belger and Brauer 2018).

The results from information seeking studies with primates and dogs collectively suggest that some of these species respond according to their own uncertainty when locating food or other objects. The highly flexible performance of apes across these types of tasks is of particular interest given the link between metacognition and Theory of Mind [see (Call 2005; Flavell 2000; Sodian et al. 2012) for discussions]. Following Premack and Woodruff’s (1978) seminal paper, a large field of research has since been dedicated to understanding what animals understand about the minds and perspectives of others, with some of the strongest evidence for these abilities emerging in the great apes [e.g., (Krupenye et al. 2016)], although much less research has focused on what animals understand about their own minds.

Outside of primates, corvids have been extensively studied for their abilities related to Theory of Mind and indeed are capable of inferring the perceptual access of others (Bugnyar et al. 2016), but less research has focused on whether and how they monitor their own knowledge states. Using a delayed match to sample task, Goto and Watanabe (2012) found that crows selected a post-trial ‘escape’ option to receive a lower-probability reward more frequently following trials in which they had chosen incorrectly, suggesting that they retrospectively monitored the strength of their memory traces. In another task, jays looked longer through peepholes when they could learn where in the adjacent compartment a food item would be hidden, compared to one in which they could locate the food regardless (Watanabe et al. 2014). Most recently, Western scrub jays were presented with a variation of Call and Carpenter’s (2001) task using two opaque tubes (Watanabe and Clayton 2016). The jays looked into the tubes more often when they had not witnessed the baiting, after a delay, and when the food had been visibly moved to another tube. The authors suggest that performance on this last condition in particular is consistent with a metacognitive account, as the birds did not immediately select where they had seen the food last but rather sought information as a result of increased uncertainty after the food was moved.

We presented the information seeking task to a group of captive ravens to determine whether they also seek information about the location of food and which search strategies they employ when doing so. Our methods were similar to those of Call and Carpenter (2001) and Call (2010): we presented the birds with three opaque tubes and compared their looking performance across trials in which they had watched the experimenter bait the tube, had no information about the location of the reward, or could infer the location about the reward by inference or using auditory cues. These latter two conditions were included to further address alternative explanations for looking behaviour; namely whether ravens use a generalized search strategy.

## Methods

### Subjects and materials

The subjects were five adult, hand-raised ravens (M:1; F:4) housed at the Corvid Cognition Station in Lund, Sweden. All subjects were housed in accordance with the regulations of the Swedish Agricultural Board (SJVFS 2019:9), and provided with ad libitum access to food and water. Participation in the experiments was entirely voluntary. For training and testing, subjects were separated in a large enclosure (roughly 100m^2^) that made up part (EM, NO, TO, RI) or all (JU) of their home enclosure. During separation subjects remained in visual and vocal contact with conspecifics. They could travel about the compartment freely and choose to visit one area of the enclosure to interact with the experimenter and the apparatus, both present in an adjacent compartment, through a mesh fence.

**Fig. 1:**
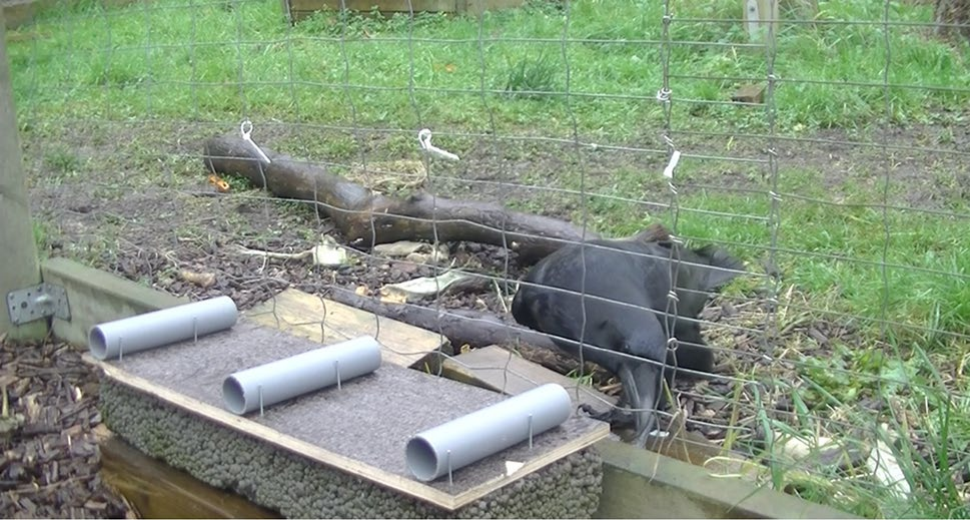
A raven lowering its head to peer inside of a tube. To select the tube, the subject must touch the ‘button’ located directly above the tube

The apparatus consisted of a wooden tray (58 × 21 cm) which could be slid across a larger block toward and away from the mesh (see Fig. 1). Three opaque tubes (18 cm long, 4 cm diameter) were placed on the tray 20 cm apart. The apparatus was 10 cm from floor level so that subjects had to conspicuously lower their heads to peer into the tubes. In the unseen test trials, a barrier (57 × 20 × 20cm) was used when baiting the tubes. Throughout testing and training three plastic cable ties (hereafter referred to as “buttons”) were fixed to the fence at 30 cm height. To select a tube, subjects were required to touch the corresponding button fixed above the tube with their beak. This was introduced so that a clear distinction could be made between selecting a tube and looking inside it.

### Training

Subjects were first trained to peck the button above a single opaque tube that contained a preferred food reward (1/4 piece of Frolic dog food). Following this, the single tube was moved randomly to the left, middle, and right positions of the board, and subjects were rewarded for contacting the button located directly above the tube with the beak. If subjects chose one of the two incorrect buttons corresponding to empty spaces on the board the experimenter pulled the tray back and then began the next trial.

After responding correctly on 5/6 consecutive trials, three tubes were placed on the board and subjects watched as the experimenter baited one of the tubes (position pseudorandomized across trials). The experimenter then pushed forward the tray to indicate the start of the trial. Once subjects selected a button the experimenter tipped the corresponding tube forward, so that the reward (or no reward if subjects chose incorrectly) would fall out of the tube and within the subject’s reach. Subjects received a maximum of 24 training trials per day and continued to testing after passing 10 of 12 consecutive trials.

### Testing

Each subject received eight sessions of 12 trials each (96 trials in total), with no more than one session per day. The final two test sessions of subject EM, which were conducted in succession over a single day, were an exception to this. Each session consisted of three of each of the following trial types, with the reward location and trial type pseudorandomized and occurring no more than twice in a row:

- Seen: The experimenter held up the reward for three seconds, conspicuously placed it inside one of the three opaque tubes, and pushed forward the tray.
- Unseen:
  Baseline: the experimenter held up the reward for three seconds, then lowered it behind a barrier placed in front of the three opaque tubes. The experimenter touched the distal end of each of the tubes from left to right, leaving the reward in one of the tubes, to account for movement and possible auditory cues. The experimenter then removed the barrier and pushed the tray forward.
  Auditory: similar to baseline trials, except after removing the barrier and just prior to pushing the platform forward, the experimenter placed one hand over each end of the tube and shook each tube (order: left, middle, right) five times in a vertical motion, so that the tube containing food provided an auditory cue.
  Inferred: similar to baseline trials, except that two of the tubes were transparent. The reward was always concealed in the opaque tube.

For all trials, the experimenter only proceeded to bait the tube, or lower the reward behind the barrier, if the subject was attending. If subjects failed to choose after 30 s the trial was repeated at the end of the session. Once subjects selected a tube by pecking the corresponding button, the tube was lifted so that any contents would fall out in reach of the subject. After subjects retrieved any rewards the tray was pulled back, the barrier placed in front of the tubes, and a new trial commenced.

### Coding

All test sessions were video recorded and coded using BORIS (Friard and Gamba 2016). Looking behaviour was coded when subjects conspicuously lowered their head to peer into the tubes (see Fig. 1). If subjects raised the head and then lowered it again to peer into the same tube this was counted as a second, discrete ‘look’. We took three dependent measures from the video: Look (as a binary variable, yes/no), frequency of looking, and location of looking (left, medium, right, or ambiguous). A portion (15%) of the videos were independently coded by an observer blind to the study’s hypothesis. Inter-observer reliability was strong (0.80).

During trials, subjects could also make different types of errors and use different search strategies. Specifically, subjects might err by selecting a tube that they had already searched and found empty, or by selecting an empty tube after seeing the reward in one of the other tubes. For search behaviour, we used definitions similar to those of Call and Carpenter (2001). Searches were coded as *insufficient* if subjects looked in only one empty tube before selecting (thereby lacking sufficient information to know the reward’s location). If subjects searched the same empty tube more than once within the same trial (excluding successive looks), this was coded as *redundant.* Finally, if subjects continued searching after already locating the reward in one of the other tubes, this was coded as an *excessive* search.

### Analyses

We focused our analysis on the number of discrete looks each subject made within a trial, which allowed us to assess not only whether subjects sought information or not, but how their patterns of information seeking varied across conditions. To estimate how much the number of looks differed between the four conditions, we fitted a Generalized Linear Mixed Model [GLMM; (Baayen 2008)] with a Poisson error structure and log link function (McCullagh and Nelder 1989). We included condition as a fixed effect, and to control for session number we included it as a further fixed effect. As random intercept effects, we included the identity of the individual and also session nested within individual to account for the number of looks varying among sessions. To keep type I error rate at the nominal level of 0.05, we included random slopes (Barr et al. 2013; Schielzeth and Forstmeier 2009) of condition (manually dummy coded and then centred) within subject and session, and of session number within subject. Originally, we also included the correlations among random intercepts and slopes. However, as most of these seemed unidentifiable as indicated by absolute correlations being close to one (Matuschek et al. 2017) we excluded them from the final model. This had a minor effect on the log-likelihood of the model (model with correlation parameters: − 697.806; model without correlation parameters: − 699.31).

We fitted the model in R (version 3.6.1; R Core Team 2019) using the function glmer of the package lme4 [version 1.1–21; (Bates et al. 2015)]. Prior to fitting the model, we z-transformed session number to a mean of zero and a standard deviation of one to ease model convergence. To test the significance of condition, we compared the full model with a reduced model lacking condition but being otherwise identical using a likelihood ratio test (Barr et al. 2013; Dobson 2002). We determined confidence intervals of the fitted model by means of a parametric bootstrap (*N* = 1000) implemented using the function bootMer. We determined model stability by excluding the levels of the random effects, one at a time and comparing the model estimates obtained for these subsets with those obtained for the entire data set. These revealed the model to be of good stability (see results). The model was not overdispersed (dispersion parameter: 0.553). The sample size for the model consisted of a total 480 trials conducted in 40 sessions with five individuals.

## Results

All subjects passed the final training step (range: 30–92 trials) and proceeded to testing. Our test data are summarized in Table 1, and detailed results from our analysis are presented in Table 2. Due to low variation in terms of looking (y/n), errors and search strategies, we provide descriptive statistics on these variables, as well as accuracy.

**Table 1:** Summary of subjects’ looking and choice responses across the four conditions

	Baseline	Auditory	Inferred	Unseen (grouped)	Seen	Total (all trials)
EM						
Percent correct (of 24)	100	88	100	96	96	96
Percent of trials looked (of 24)	100	100	100	100	96	99
Average looks/trial	2.46	2.46	1.58	2.17	1.78	2.07
JU						
Percent correct (of 24)	71	54	92	72	75	73
Percent of trials looked (of 24)	96	96	96	96	79	92
Average looks/trial	1.91	1.78	1.30	1.67	1.05	1.53
NO						
Percent correct (of 24)	75	41	88	68	83	72
Percent of trials looked (of 24)	100	83	88	90	96	92
Average looks/trial	2.00	2.35	1.29	1.88	1.26	1.72
RI						
Percent correct (of 24)	88	88	92	89	92	90
Percent of trials looked (of 24)	96	100	92	96	96	96
Average looks/trial	2.30	3.04	1.41	2.25	1.52	2.09
TO						
Percent correct (of 24)	83	71	92	82	96	85
Percent of trials looked (of 24)	100	92	100	99	100	99
Average looks/trial	2.40	3.00	1.13	2.18	1.75	2.05
Grand total						
Percent correct (of 24)	83.4	68.4	92.8	81.4	88.4	83.2
Percent of trials looked (of 24)	98.4	94.2	95.2	96.2	93.4	95
Average looks/trial	2.22	2.53	1.34	2.03	1.47	1.89

**Table 2:** Results of the GLMM about the effect of condition on the number of looks (estimates, together with standard errors, confidence limits, significance test, and range of model estimates obtained after excluding levels of the random effects one at a time)

Term	Estimate	SE	Lower Cl	Upper Cl	*χ*^2^	*df*	*P*	Min	Max
(Intercept)	0.755	0.086	0.582	0.907			^a^	0.717	0.805
Condition: auditory^b^	0.119	0.085	− 0.031	0.280	25.630	3	< 0.001	0.059	0.153
Condition: inferred^b^	− 0.523	0.102	− 0.705	− 0.330				− 0.562	− 0.464
Condition: seen^b^	− 0.429	0.099	− 0.607	− 0.234				− 0.463	− 0.383
z.Session^c^	− 0.063	0.034	− 0.129	0.003	3.181	1	0.075	− 0.092	− 0.042

^a^Not indicated because of having a very limited interpretation

^b^Dummy coded with baseline being the reference category; the indicated test refers to the overall effect of condition

^c^z-transformed to a mean of zero and a standard deviation of one; mean and sd of the original variable were 4.500 and 2.294, respectively

### Looking by trial type

**Fig. 2:**
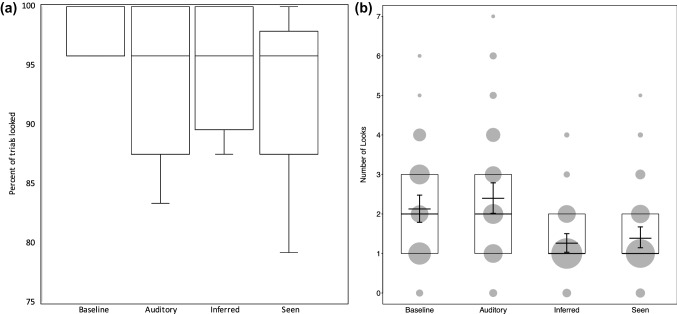
**a** Percent of trials in each condition in which subjects looked into at least one tube. Boxes and wide horizontal lines indicate medians and quartiles, and short horizontal lines indicate minimum and maximum values. **b** Number of looks, separately per condition. Thick horizontal lines and boxes indicate medians and quartiles, and short horizontal lines with error bars indicate the fitted model and its confidence interval. The area of the dots is proportionate to the number of trials per combination of condition and number of looks (range: 1–82)

As a group, subjects looked inside at least one tube on the majority of trials (Median: 96%) and across the different trial types (Fig. 2a). Overall, we found clear differences between conditions with regard to the number of looks within trials, whereby individuals made more discrete looks in the baseline and auditory conditions as compared to the inferred and seen conditions (Table 2; Fig. 2b).

### Accuracy by trial type

Subjects chose correctly on the majority of trials (roughly 85% percent of all trials), including 92% of seen trials and 82% of all unseen trials. Of the trials where subjects chose incorrectly, they still looked into at least one of the tubes on 82% of trials (range: 60–100%).

### Errors

On incorrect trials, subjects could make errors in several different ways, including by selecting a tube that they had already seen was empty, or selecting an empty tube after they had already looked into the tube containing the reward. The most frequent error involved selecting a tube that they had seen was empty (Median: 11% of trials, range: 3–14%), and on a few instances, subjects selected an empty tube after seeing the reward in one of the other tubes (M: 2%, range: 0–2%). On other trials, subjects made both errors (M: 3% of all trials, range: 1–3%). The number of these occurrences was too small to analyse across conditions per individual.

### Search strategies

Subjects used an efficient search strategy, in which they searched for the food until they located it, on the majority of trials (M: 81%, range: 74–85%). Subjects used a redundant search strategy, in which they looked into the same empty tube more than once, on a median of 5% of trials (range: 1–12%) and an inefficient search strategy, in which they looked into only one empty tube before choosing, on a median of 3% of trials (range: 3–15%). Excessive searches, where subjects continued to search in the other tubes after already seeing the reward, occurred on 5% of trials (range: 3–12%). We further detail differences in search strategies across conditions in Table 3.

**Table 3:** Median proportion of trials (range in parentheses) in which subjects used the different search strategies for each condition (*N* = 24 trials per condition)

	% Auditory	% Baseline	% Inferred	% Seen
Efficient	67 (46–75)	79 (58–88)	96 (92–96)	88 (79–96)
Inefficient	4 (4–20)	13 (8–17)	4 (4–4)	13 (8–17)
Redundant	15 (4–25)	4 (5–17)	4 (4–4)	6 (4–8)
Excessive	8 (4–21)	8 (4–8)	4 (4–4)	4 (4–4)

*Efficient* searching until locating the food; *inefficient* searching inside only one empty tube before choosing; *redundant* looking inside the same tube more than once; *excessive* continuing to search the tubes after locating the food

## Discussion

Overall, the ravens located and selected the correct tube on the majority of trials. Most notably, subjects also looked inside at least one tube on nearly every trial, including those in which they had either witnessed the baiting, or could use visual or auditory clues to infer the reward’s location. However, on seen and inferred trials, subjects tended to look only once, inside the baited tube, whereas they looked a greater number of times, indicative of searching behaviour, within auditory and hidden trials.

The ravens’ search behaviour deviates from that of chimpanzees, orangutans, and rhesus macaques, which looked significantly less on seen compared to hidden trials (Call 2010; Call and Carpenter 2001; Hampton et al. 2004; Marsh and MacDonald 2012). Instead their performance is most similar to capuchins in a study by Basile and colleagues (2009). In the first experiment, the capuchins also showed ceiling effects of looking, however their first looks were often directed toward an incorrect tube, suggesting that they were not attentive to the baiting on seen trials. Following a training in which subjects were required to attend to the baiting, some of the capuchins then looked significantly less on seen trials than unseen trials (Experiment 2). In the case of the ravens, however, subjects’ directed searches toward the correct tube indicate that they had indeed been attending to the baiting. Furthermore, the birds spontaneously demonstrated this searching behaviour during the final step of their training, which was similar to the seen condition in the test (see also Basile et al. 2009).

In addition to the seen trials, the ravens continued to search on trials in which they had not witnessed the baiting, but could either infer the reward’s location, or were provided with auditory clues. One explanation for their continued searching on auditory trials is that they lacked the necessary experience using auditory cues to solve a task. For example, Call (2010) found that only those chimpanzees that had previously learned to use auditory information looked less on trials when the tubes were shaken, whereas those that had not continued to look across trials. Indeed, unlike seen trials or inferred trials, subjects searched the tubes randomly, suggesting that they were not using auditory cues to guide their search.

The ravens also continued to search when presented with only one possible location for the reward (i.e., two empty, transparent tubes and one opaque tube). Their performance is comparable to that of capuchin monkeys and lion-tailed macaques, which similarly continued to search on trials in which they could infer the reward’s location (Marsh 2014; Paukner et al. 2006; Vining and Marsh 2015), and contrasts with other species such as orangutans which tended to look less on inferred trials (Marsh and MacDonald 2012). As previous studies have shown that ravens are capable of inferring by exclusion (Schloegl et al. 2009), and given that they also looked on the majority of seen trials, this likely reflects their general tendency to search on all trials, even if they were aware of where the food was. Indeed, when searching the tubes, subjects tended to look only once inside the baited tube, as they also did on seen trials, before choosing.

This efficient search strategy, in which subjects continued to look inside the tubes until the reward was located, is similar to that of the chimpanzees tested by Call and Carpenter (2001). On very few trials, the ravens searched excessively by continuing to check the tubes after they located the reward, or they used an insufficient search strategy in which they looked inside only one empty tube before selecting. Unlike several of the chimpanzees, however, which continued looking in the tubes after locating the reward on nearly half of all trials, ravens did so on very few (5%) of the trials, typically stopping and selecting the correct tube once they located the food. Similarly, the children tested by Call and Carpenter (2001) showed excessive searches on roughly 35–40% of trials. Unlike the chimpanzees, however, the ravens rarely terminated their searches early, for example after looking into two empty tubes. In this way they also differ from the jays, which tended to select the remaining tube without searching it, if they had previously searched the other tube (Watanabe and Clayton 2016).

There are several potential explanations for the ceiling levels of searching shown by the ravens. In particular, their targeted searches into the baited tube on seen and inferred trials may reflect what Call (2010; Call and Carpenter 2001) termed the ‘passport effect’, in which the benefit of double-checking the tube’s contents is lower than the cost. Though the buttons were designed to separate looking and selecting such that the ravens had to discretely lower their heads to peer into the tubes and raise their heads to make a choice, it appears that the cost of checking was still low. In addition, we used a highly preferred food reward. These two conditions would provide an ideal scenario for the passport effect; however, this needs to be confirmed with further research manipulating the reward type and cost of searching. One future means of imposing a greater cost of searching might be to use a titration procedure similar to that used in other studies in which the tubes are gradually lowered until subjects reach a threshold criterion of looking that avoids ceiling or floor effects (e.g., Hampton et al. 2004). As such a setup is difficult due to the ravens’ size and morphology, an alternative solution would be to require the birds to exert more effort, for example by opening a door (which could then vary in difficulty to open) to peer inside a compartment before selecting (similar to Basile et al. 2009).

The birds’ performance could also reflect a tendency to look inside the tubes because the sight of the food is rewarding in itself, rather than with the goal of gaining information about the reward’s location. As has been discussed elsewhere (Call 2012), however, it would not explain why, upon locating the food, the birds then chose rather than looking inside the tube again. Additionally, the ravens showed behaviour consistent with searching for a specific item (e.g., rarely searching the same tube twice) rather than peering randomly inside the tubes until they saw the reward, as might be predicted otherwise. Nonetheless, it would be of interest to test corvids’ information seeking behaviour with non-food items, as has been done with apes (Bohn et al. 2017), to determine whether this generalizes to non-foraging contexts.

Finally, that the ravens looked across seen trials runs counter to the predictions of the generalized search response, or the response competition hypothesis, which both suggest that subjects would be more likely to immediately select the baited tube after having seen the reward placed there, rather than looking inside it (Carruthers 2008; Hampton et al. 2004; Perner 2012). As the ravens had learned to use the buttons to select a tube, and did not attempt to reach through the fence with the beak but rather lowered their heads to briefly peer inside, their search responses on seen trials are also unlikely to reflect any type of automatic reaching response. Additionally, as the delay between baiting and selecting was short and the birds tended to look only inside the baited tube, it is unlikely that they checked as a result of forgetting.

It may be that the frequent checking behaviour demonstrated by ravens relates to their food-caching and cache pilfering strategies. In addition to caching their own food, ravens often pilfer the caches of conspecifics, and experience their own caches being pilfered. As such they demonstrate sensitivity to the presence and visual access of others, often taking measures to protect their caches, such as relocating them if they have been watched, or creating ‘false’ caches (Bugnyar and Kotrschal 2002; Heinrich and Pepper 1998). In these false caching events, individuals behave as though they are creating a cache but the cache remains empty, either because they have not deposited food into it or because they have inconspicuously taken the food back out of the cache. Consequently, pilferers often face situations in which they have witnessed food being placed in one location only to find it empty. Such experience may explain why, despite the experimenter conspicuously placing the reward into the tube, subjects chose to look first inside the tube before selection. This might be tested by presenting the same paradigm to ravens that lack this experience (e.g., young ravens, or those that have been hand reared without conspecifics).

This study provides first comparative data on the performance of ravens in an information seeking task typically used to measure metacognitive abilities in other species. We found that when presented with varying degrees of information about which tube a food item was hidden in, the ravens chose to look inside the tubes on virtually every trial, including those in which they had seen where the food was placed. However, on trials in which they had witnessed the baiting or could infer the reward’s location, the ravens tended to look only once inside the correct tube, whereas they looked more often within baseline and auditory trials. Considering the high frequency of looking in this task overall, an important next step is to identify the conditions under which ravens are likely to change their search behaviour, so that differences across trial types can be detected. Such future studies are likely to be particularly illuminating given the frequent uncertainty these birds face during cache retrieval, as well as their performance in tasks measuring Theory of Mind and related cognitive abilities.

## Electronic supplementary material

Below is the link to the electronic supplementary material.

Supplementary file1 (XLSX 30 kb)
